# Impact of adherence to six-month home-based HIIT on cardiorespiratory fitness in older adults

**DOI:** 10.1186/s13102-026-01750-5

**Published:** 2026-05-14

**Authors:** Sindre Herskedal Fosstveit, Jack Feron, Hilde Lohne-Seiler, Kelsey E. Joyce, Katrien Segaert, Samuel J.E. Lucas, Sveinung Berntsen

**Affiliations:** 1 Department of Sport Science and Physical Education, Faculty of Health and Sport Sciences, University of Agder, Kristiansand, Norway; 2 School of Sport, Exercise and Rehabilitation Sciences, University of Birmingham, Birmingham, UK; 3 Centre for Human Brain Health, University of Birmingham, Birmingham, UK; 4 School of Psychology, University of Birmingham, Birmingham, UK; 5 Research Unit, Sorlandet Hospital, Kristiansand, Norway

**Keywords:** Exercise volume, High-intensity, Lactate threshold, Senior, Session duration, Trainability, Training frequency, V̇O_2PEAK_

## Abstract

**Background:**

High-intensity interval training (HIIT) was originally designed to improve athletic performance, but a growing body of research over the past decade has highlighted its positive impact on various other health outcomes. However, concerns exist regarding HIIT’s suitability for those unaccustomed to regular exercise, as its high intensity may impact tolerance and adherence. Therefore, this study aimed to assess the associations between various objective adherence metrics and the resultant changes in peak oxygen uptake (V̇O_2PEAK_) and lactate threshold (LT) in older adults completing six-month home-based HIIT.

**Methods:**

Healthy older adults (*n* = 233, 60–84 years, 54% female) were randomized to six-month, thrice-weekly home-based HIIT or a passive control group. Adherence in the HIIT group was objectively monitored using a Polar watch and heart rate sensor and quantified as frequency (sessions/week), intensity (minutes ≥ 80% HR_PEAK_/session), duration (session duration, min), and total adherence (cumulative MET-min; overall exercise volume summed across sessions from session duration and mean %HR_PEAK_). For each metric, adherence was expressed as a percentage of completion relative to the planned amount. V̇O_2PEAK_ and LT were assessed using a modified Balke treadmill protocol to volitional exhaustion. To account for multicollinearity, partial least squares regression (PLSR) models assessed associations between adherence metrics and changes in V̇O_2PEAK_ or LT.

**Results:**

The PLSR analysis, accounting for baseline V̇O_2PEAK_, age, sex, and country where data-collection took place, revealed a positive association between post-test V̇O_2PEAK_ and total adherence (Selectivity fractions (SF) = 0.70 [0.19; 0.93]), frequency adherence (SF = 0.54 [0.08; 0.86]), and intensity adherence (SF = 0.44 [0.07; 0.80]), but not with duration adherence (SF = 0.11 [− 0.08; 0.54]). For LT, PLSR analysis revealed no associations with any adherence metrics.

**Conclusions:**

Superior HIIT adherence (i.e., total, frequency, and intensity) was associated with larger V̇O_2PEAK_ gains in older adults. Notably, total adherence demonstrated the strongest predictive contribution among the adherence metrics, suggesting that overall exercise volume may be especially relevant for improving V̇O_2PEAK_ in this context. In contrast, adherence metrics did not predict changes in LT, despite the exercise group experiencing significant LT improvements, indicating that LT adaptations were not strongly explained by variation in HIIT adherence in this sample.

**Trial registration:**

ClinicalTrials.gov NCT07443189 (Registration date: 02.03.2026, retrospectively registered).

## Background

High-intensity interval training (HIIT) has gained attention as an alternative to moderate-intensity continuous training (MICT), providing comparable health benefits in a more time-efficient manner [1], and has demonstrated positive health effects across diverse populations [2]. However, concerns have been raised about HIIT’s suitability for individuals unaccustomed to regular exercise, as its high intensity may negatively impact tolerance and adherence [3, 4].

In this context, adherence, the extent to which participants follow the prescribed frequency, intensity, time, and type (FITT), is central to efficacy and health outcomes [5]. Traditional adherence metrics (e.g., attendance or dropout) overlook crucial elements such as exercise intensity, limiting subsequent interpretation, reproducibility, and synthesis of findings [6–9]. Accurate, objective adherence assessment is therefore essential, particularly in home-based programs where reliance on self-report introduces recall bias [10, 11]. Furthermore, studies comparing HIIT and MICT find that some HIIT participants fail to reach target intensities, while some MICT participants exceed prescriptions [12, 13], highlighting the need to objectively quantify the actual exercise volume performed, as variations in adherence could explain variations in key outcome measures (e.g., cardiorespiratory fitness (CRF)) [14, 15].

Moreover, there is considerable interindividual variability (“trainability”) in CRF responses to a given training stimulus, largely influenced by genetics [16]. However, individuals with minimal peak oxygen uptake (V̇O_2PEAK_) improvements may still improve other CRF-related variables, such as lactate threshold (LT) (i.e., the intensity of exercise at which the accumulation rate is faster than the removal rate of blood lactate concentration ([La^−^]_b_)) [17]. Accordingly, focusing solely on V̇O_2PEAK_ may understate meaningful physiological gains to exercise training, as complementary outcomes like LT capture submaximal and muscular adaptations that V̇O_2PEAK_ alone may not reflect [17, 18]. Given the broad health benefits associated with higher CRF [19–23], it is important to identify which aspects of an exercise intervention (e.g., frequency, duration, intensity, or volume) most strongly influence CRF in order to tailor exercise interventions effectively.

Regular exercise is critical for healthy aging [5] and, therefore, innovative methods are essential to accurately capture the exercise behaviors of older adults, particularly in non-controlled (e.g., home-based) settings [24]. Adherence in such settings is challenged by the difficulty of self-monitoring prescribed exercise intensity without direct supervision [24] and by environmental factors such as terrain, weather, and outdoor safety [25]. Understanding these behaviors is fundamental for tailoring exercise interventions to the unique needs and challenges of older populations, potentially enhancing health outcomes and quality of life [26]. Although our recent six-month, home-based, HIIT trial showed high adherence and significant improvements in V̇O_2PEAK_ and LT [27], there was substantial interindividual variability [27], and it remains unclear which aspects of adherence best explain this variability.

Therefore, the present study links objectively assessed adherence (frequency, time in target intensity, session duration, and total volume) to changes in V̇O_2PEAK_ and LT in older adults completing a six-month, home-based HIIT intervention. We aim to inform the practical prescription and monitoring of guideline-based exercise to help older adults achieve meaningful CRF gains in real-world settings. We hypothesized that V̇O_2PEAK_ and LT adaptations would show broadly similar associations with the adherence metrics, given that the present home-based HIIT protocol was previously shown to produce significant improvements in both outcomes [27]. Moreover, we expected some differences in magnitude, with intensity adherence more strongly associated with V̇O_2PEAK_ and session duration more strongly associated with LT.

## Methods

### Study design

This study was part of the “Fitness, Ageing, and Bilingualism” (FAB) project (see OSF preregistration: osf.io/6fqg7). Participants were screened online (Physical Activity and Health Pre-screening Questionnaire (see supplementary materials), Language Experience and Proficiency Questionnaire [28]), and eligible participants were randomized to a home-based exercise group or a control group stratified by age and sex using a bespoke algorithm. Allocation was generated individually after assignment of a participant ID and was not predictable in advance. Participants were informed of group allocation at the first in-person visit. Participants completed a standardized graded maximal exercise test to exhaustion at pre- and post-intervention (26 weeks) to assess V̇O_2PEAK_ and LT. The exercise group was prescribed three weekly sessions (unsupervised), supported by coaches through monthly emails, phone calls, and face-to-face meetings. Target training intensity during high-intensity intervals was > 80% of peak heart rate (HR_PEAK_), determined during V̇O_2PEAK_ testing. Because the same study team was responsible for the supervised familiarization, ongoing participant follow-up, and the pre- and post-intervention maximal exercise tests, outcome assessors were not blinded to group or adherence status; the potential implications of this design feature are addressed in the limitations.

### Participants

**Fig. 1:**
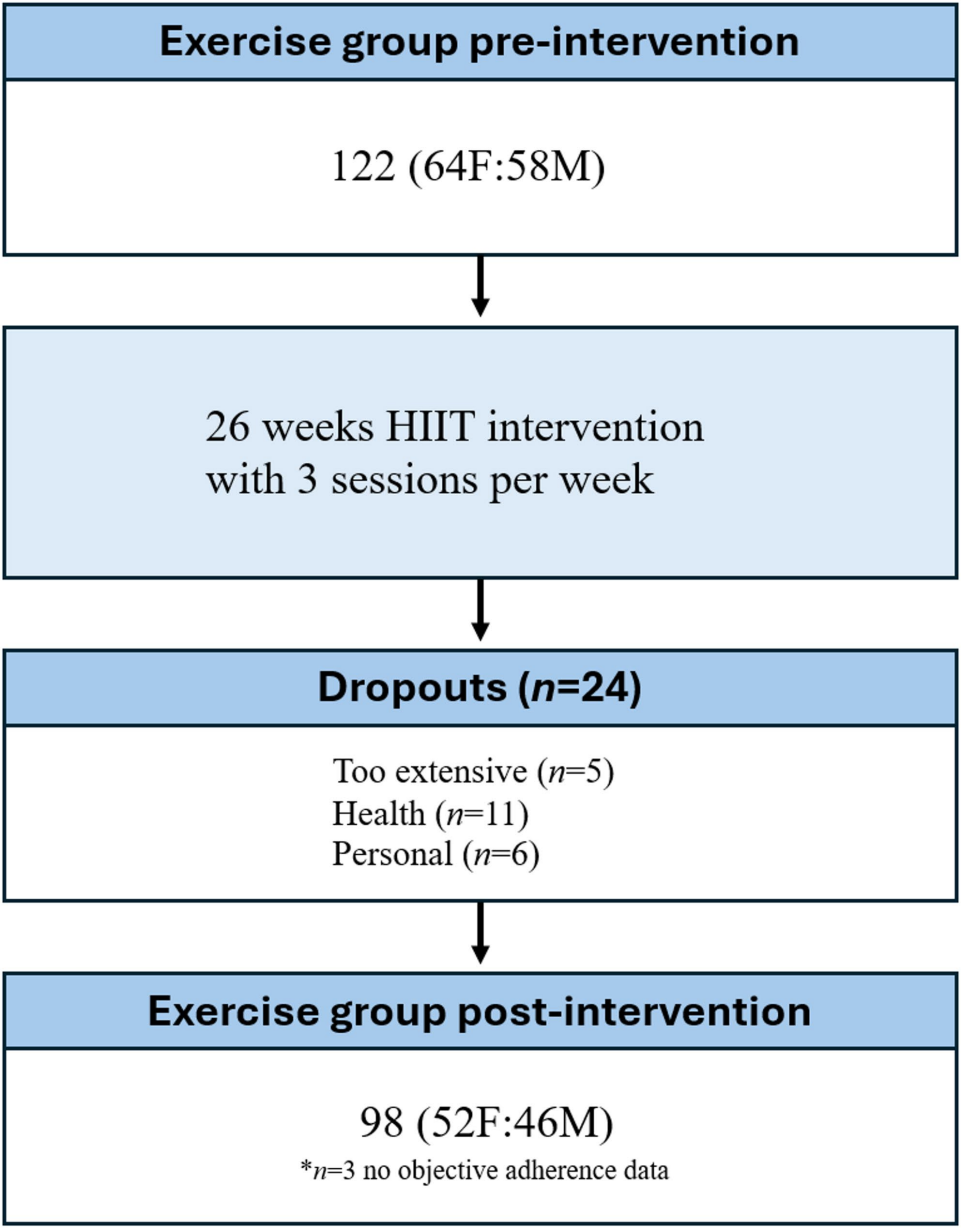
Study design flowchart illustrating the timeline for the exercise group from pre- to post-intervention. *Abbreviations*: *F* Female, *HIIT* High-intensity interval training, *M* Male, *n* Number

The study recruited 233 healthy, home-dwelling older adults aged 60–84 years (67 ± 6 years, 54% female) from southern Norway (*n* = 139) and the West Midlands, UK (*n* = 94), between September 2021 and November 2023. Of these, 122 were randomized into the exercise group and completed 26 weeks of home-based HIIT (68 ± 6 years, 52% female). Among the 122 participants randomly assigned to the exercise group, 98 completed the intervention, with 95 providing objective adherence data (Fig. 1). The remaining three completers were excluded from adherence analyses because objective Polar heart-rate data were unavailable, owing to device malfunction or the device not being worn during sessions. Participants were non-smokers for ≥ 5 years, had normal or corrected-to-normal vision and hearing, free of language impairments and significant health conditions, and self-reported low physical activity levels (< 150 min/week of moderate-intensity exercise). Norwegian participants provided a health certificate from their GP, while UK participants with severe ECG abnormalities or hypertension were excluded. These criteria aimed to select inactive but otherwise healthy older adults across both sites.

### Ethics statement

This study adhered to the Declaration of Helsinki, and data storage followed Norwegian regulations (Norwegian Agency for Shared Services in Education and Research, ref.: 239577). The project was approved by the Regional Committee for Medical and Healthcare Research Ethics in Norway (REK, ref.: 163931) and received institutional ethical approval from the University of Agder (The Ethical Board at the Faculty of Health and Sport Science) and the University of Birmingham (ERN 20_1107). The present study was conducted in accordance with the CONSORT 2025 guidelines for reporting randomized controlled trials.

Prior to study entry, participants received a detailed information sheet describing the study structure, inclusion criteria, confidentiality procedures, and their rights regarding participation and withdrawal. After reviewing the information, written informed consent was obtained from all participants who agreed to take part.

### Exercise intervention

The first four weeks served as a familiarization period, introducing participants to the program and equipment, including the Polar Unite watch, Polar H9 sensor (Polar Electro Oy, Kempele, Finland), and training logbook. The Polar watch, preprogrammed to show %HR_PEAK_, enabled real-time monitoring of FITT factors during exercise sessions. Personal coaches (SHF, JF) provided guidance with weekly follow-ups during the familiarization period, which progressed to monthly thereafter. At each monthly contact, coaches reviewed the participant’s Polar heart-rate session data and training logs; when a participant was consistently below the > 80% HR_PEAK_ intensity target during high-intensity intervals, the shortfall was discussed and verbal encouragement was provided to raise intensity on subsequent sessions. Between contacts, the Polar watch’s real-time %HR_PEAK_ display served as the primary within-session intensity cue.

Each session, lasting 40–60 min, included a warm-up, the main exercise protocol, and a cool-down. Participants were prescribed one circuit and two interval training sessions each week. Circuit training sessions consisted of six exercises, each performed for 45 s with 30-second rest periods between sets. Interval training consisted of 2-minute high-intensity intervals with 2-minute active recovery between each, primarily as uphill walking. The number of intervals gradually increased from five to ten intervals throughout the program. Participants were asked to aim for a heart rate > 80%HR_PEAK_ during each high-intensity interval/circuit set and complete as many repetitions as possible of each circuit exercise. A comprehensive description of the exercise intervention used in the present study is available in our prior publication [27]. To support safe execution of the home-based sessions, the familiarization period was supplemented by pre-recorded video demonstrations of each circuit movement with explicit scaling alternatives. Warm-ups were session-specific: approximately 5–10 min of dynamic lower- and upper-limb drills prior to circuit sessions, and approximately 10 min at 57–70% HR_PEAK_ prior to interval sessions. Moreover, alternative modalities (e.g., rowing ergometer, cycle ergometer, cross-trainer) were permitted when minor muscle or joint concerns arose.

### Measures

#### Cardiorespiratory fitness

To assess V̇O_2PEAK_ and LT, a modified Balke treadmill protocol was used [29]. Respiratory gases (V̇O_2_ and V̇CO_2_) were recorded continuously using a facemask (Hans Rudolph, Kansas, USA) and Vyntus CPX metabolic cart (Vyaire, Illinois, USA). Blood lactate ([La−]_b_) was analyzed at the end of each stage using the EKF Biosen lactate analyzer (EKF Diagnostics, Cardiff, UK). Participants warmed up with self-paced walking, progressing to a standardized walking protocol until volitional exhaustion. Participants completed 4-minute walking stages at increasing gradients until LT was reached. Following LT, treadmill speed and/or gradient were increased every 1 min until volitional exhaustion. Heart rate was continuously measured with a Polar H9 sensor in Norway and a 12-lead ECG (Cardiosoft, Vyaire, USA) in the UK.

V̇O_2PEAK_ was calculated as the mean of the two highest 30-second readings, and LT was defined as a [La−]_b_ increase of 2.1 mmol/L above the mean of the two lowest exercising values [30]. A minimum of three treadmill stages was required to complete the sub-maximal test and establish the LT. Changes in LT were calculated as the difference (post-pre) in [La−]_b_ at the treadmill stage corresponding to LT at pre-intervention (e.g., pre-intervention LT occurred at stage 4, where [La−]_b_ = 3.5 mmol/L, post-intervention stage 4 [La−]_b_ = 3.0 mmol/L, and thus ∆LT = -0.5 mmol/L).

#### Exercise intervention adherence

Adherence to the intervention was calculated using only data from post-familiarization exercise sessions (i.e., weeks 5–26), excluding warm-ups and cool-downs. Heart rate data from each session was visually inspected to ensure quality control and eliminate measurement errors. The availability of sessions with usable heart rate data was recorded at 96 ± 6% among all participants in the exercise group. Sessions were classified as interval, circuit, or alternative, with the latter category including any non-prescribed activities such as rowing, hiking, or continuous aerobic exercises. A novel method based on metabolic equivalents (METs) was used to calculate adherence [6]. Planned cumulative MET-minutes (MET-mins) for the intervention period were calculated by summing the planned MET-mins for each session. Planned MET-mins were estimated by multiplying the planned session duration by the MET code corresponding to 70%-76% of HR_PEAK,_ based on the American College of Sports Medicine (ACSM) guidelines [31]. Mean heart rate for each session (including recovery bouts) was estimated to fall within this range. Cumulative MET-mins were then calculated for each participant using the actual duration and mean %HR_PEAK_ of each completed session. Total adherence (cumulative MET-mins) represents overall exercise volume. It is calculated at the session level as: MET-mins/session = (session duration in minutes) × (MET code corresponding to the session’s mean %HR_PEAK_), and then summed across all sessions over the intervention period. Example: If a participant completes a 30-minute session and the session’s mean %HR_PEAK_ corresponds to a MET code of 4.4 (per ACSM mapping), that session contributes 30 × 4.4 = 132 MET-mins to total adherence; total adherence is the sum of these MET-min values across all completed sessions. Adherence to cumulative MET-mins was the primary adherence measure, but adherence to exercise intensity (i.e., minutes > 80%HR_PEAK_ each session), session frequency (i.e., average weekly sessions), and session duration were also calculated. For all metrics, adherence was expressed as the percentage completed relative to what was planned.

### Statistical analyses

The sample size rationale for the trial has been reported previously [27]. Statistical analyses were conducted using RStudio (RStudio Team, 2021) and IBM SPSS Statistics 29 for Windows (IBM Corp., Armonk, NY, USA). Figures were created in RStudio (RStudio Team, 2021) and Prism 8.0.1 (GraphPad Software, San Diego, CA, USA).

Partial least squares regression (PLSR) models were run in RStudio using the *mvpa* package (https://github.com/liningtonlab/mvpa) [32]. To assess the individual impact of adherence metrics on V̇O_2PEAK_ and LT, models included all four adherence metrics as explanatory variables. Unlike multiple linear regression, PLSR can handle multicollinearity between explanatory variables. Separate models were run with either post-test V̇O_2PEAK_ or post-test LT as the outcome variable. Selectivity fractions (SF) with 95% confidence intervals are reported, a measure of explained predictive variance for the explanatory variables, varying between − 1 and + 1. Prior to PLSR, all variables were centered and standardized to unit variance and adjusted for relevant covariates (baseline V̇O_2PEAK_ or LT, age, sex, and country of residence). ∆LT is used as the natural descriptive quantity for characterizing the downward shift of the lactate curve, whereas the inferential PLSR and multiple linear regression models use post-test LT as the outcome with baseline LT as a covariate. Monte Carlo resampling with 1000 repetitions was used to cross-validate PLSR models and obtain a single predictive PLS component (50% of participants were used as an external validation set when estimating the models). Because PLSR reduces a potentially collinear predictor space to a small number of latent components, sample-size adequacy is more appropriately judged by retaining only the components supported by cross-validation and by predictive validation than by a fixed subjects-per-variable heuristic [33–36]. Analyses were conducted using a complete-case approach; participants with missing data for the variables required for a given analysis were excluded from that analysis.

## Results

### Study participants

The baseline characteristics of participants who completed the intervention in the exercise group are shown in Table 1.

**Table 1:** Exercise group participant characteristics at baseline

Variables	Exercise Mean ± SD
*n*	98 (52 F:46 M)
Age (years)	67.4 ± 5.7
BMI (kg/m^2^)	27.2 ± 3.6
Body mass (kg)	80.4 ± 13.4
V̇O_2PEAK_ (mL/kg/min)	27.9 ± 5.3

Values are presented as mean ± SD. *Abbreviations: BMI* Body mass index, *F* Female, *kg* Kilogram, *kg/m*^2^ Kilograms per square meter, *M* Male, *min* Minutes, *ml* Milliliter, *n* Number, *SD* Standard deviation, *V̇O*_2PEAK_ Peak oxygen uptake

### Exercise intervention adherence

**Fig. 2:**
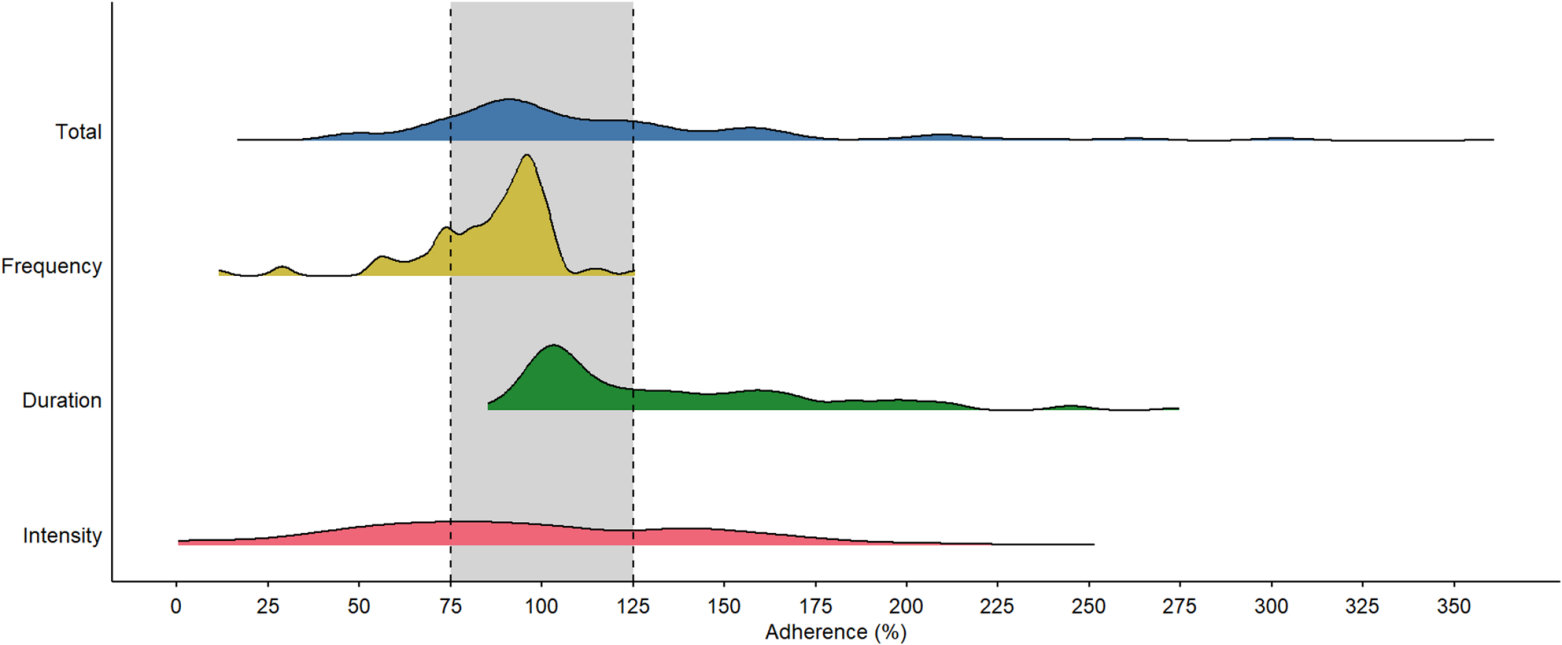
Distribution of the adherence metrics to the exercise intervention

An overview of the participants’ overall adherence (cumulative MET-mins) to the intervention, as well as specific adherence rates to frequency, intensity, and duration, is shown in Table 2; Fig. 2.

**Table 2:** Adherence to the exercise intervention

Adherence metric	Planned	Completed	Adherence (%)
Total adherence	8567	11,116 ± 5455	122 ± 59 ^a^
*Cumulative MET-mins*			
Frequency	3.0	2.6 ± 0.6	86 ± 18
*Sessions/week*			
Intensity	10.5	10.3 ± 5.2	98 ± 50
*Minutes > 80%HR*_*PEAK*_ */session*			
Duration	29.5	39.9 ± 11.8	135 ± 40
*Session duration (minutes)*			

Values are means ± standard deviations. Adherence was calculated as the percentage completion relative to what was planned for each metric. *Abbreviations*: *HR*_PEAK_ Peak Heart Rate, *MET* Metabolic Equivalent of Task. ^a^ By accounting for extended intervention periods due to sickness, minor injuries, or scheduling constraints, the calculation yields a percentage that is slightly lower than what a direct ratio of achieved to planned doses would produce

### Associations between V̇O_2PEAK_ and adherence

**Fig. 3:**
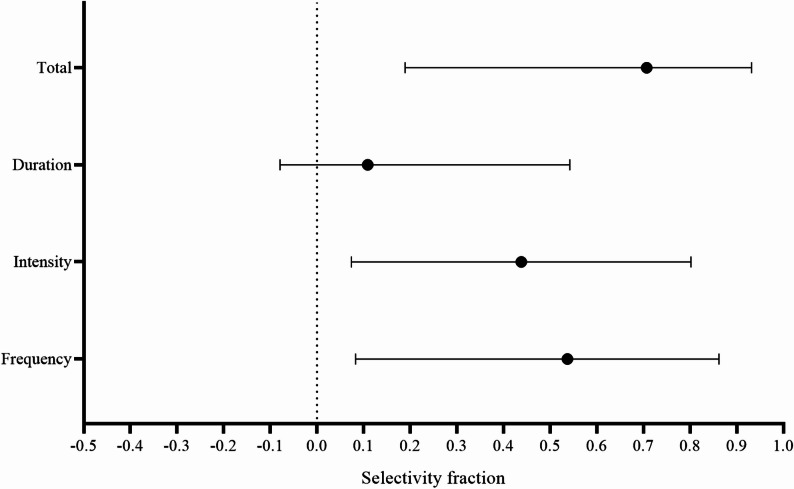
Selectivity fractions, a measure of explained predictive variance, between adherence metrics and post-test V̇O_2PEAK_. Values are medians with 95% confidence intervals. Determined using partial least squares regression

**Fig. 4:**
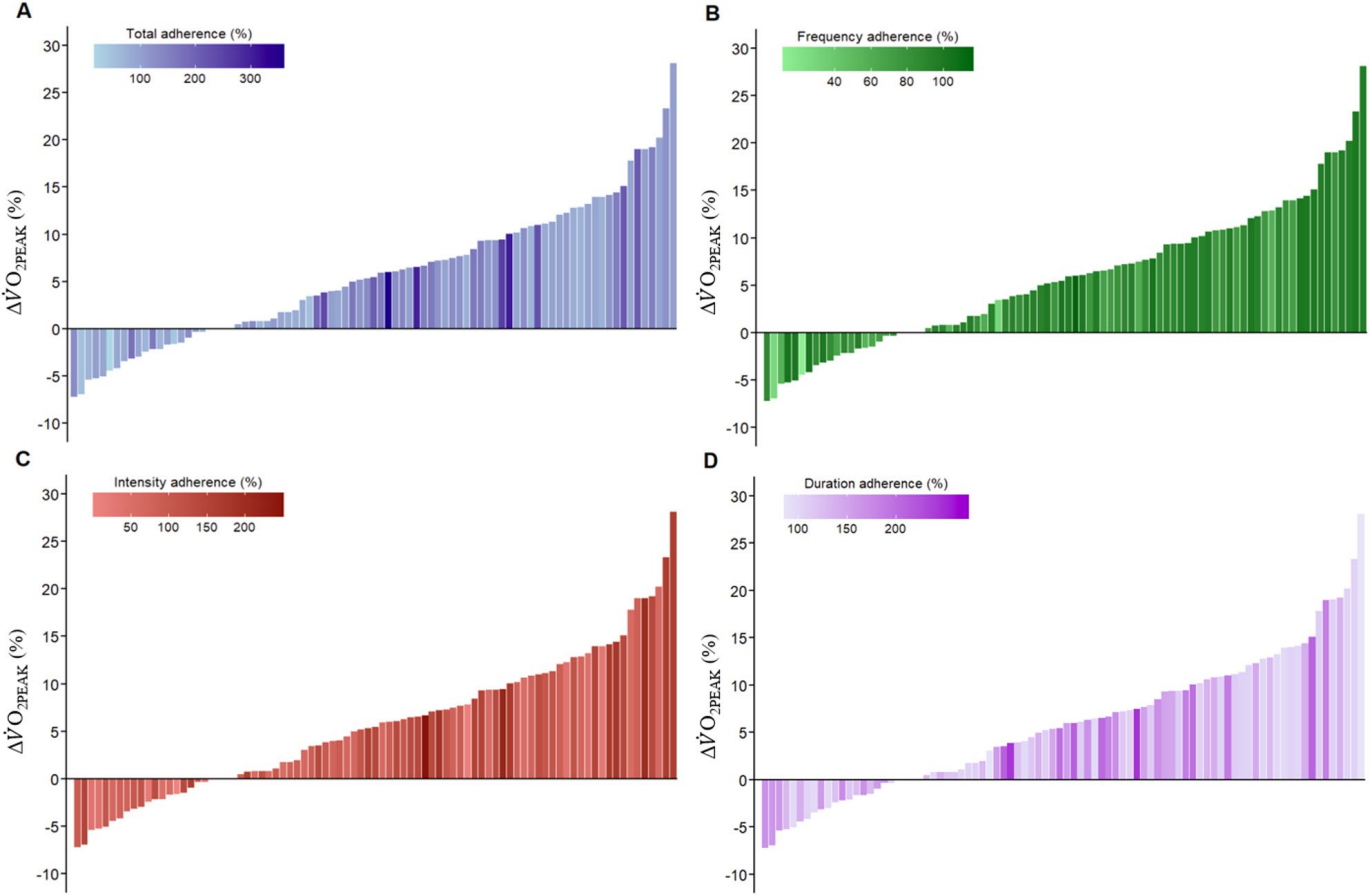
Individual participant percentage changes in V̇O_2PEAK_ in response to the six-month exercise intervention, with colors representing each individual’s (**A**) total adherence, (**B**) frequency adherence, (**C**) intensity adherence, and (**D**) duration adherence. Color-bar legends in the top-left of each panel map the gradient to numerical adherence values (%); darker colors indicate higher adherence levels

From pre- to post-intervention, V̇O_2PEAK_ increased from 28.0 mL/kg/min (95% CI 26.9–29.2) to 29.4 mL/kg/min (95% CI 28.3–30.5), a mean change of + 1.4 mL/kg/min (95% CI 1.0–1.8) or + 5.5% (95% CI 3.8–7.1%). Fifty-one of 86 participants (59.3%) achieved an increase of ≥ 1 mL/kg/min in V̇O_2PEAK_, a magnitude previously associated with clinically meaningful reductions in cardiac mortality risk [37]. The PLSR analysis, accounting for baseline V̇O_2PEAK_, age, sex, and country of residence, revealed positive associations between post-test V̇O_2PEAK_ and total adherence (SF = 0.70, 95% CI: 0.19; 0.93), frequency adherence (SF = 0.54, 95% CI: 0.08; 0.86), and intensity adherence (SF = 0.44, 95% CI: 0.07; 0.80) (Fig. 3). There was no association between post-test V̇O_2PEAK_ and duration adherence (SF = 0.11, 95% CI: −0.08; 0.54) (Fig. 3). See Fig. 4 for individual participant changes in %V̇O_2PEAK_ in response to the six-month exercise intervention.

### Associations between LT and adherence

**Fig. 5:**
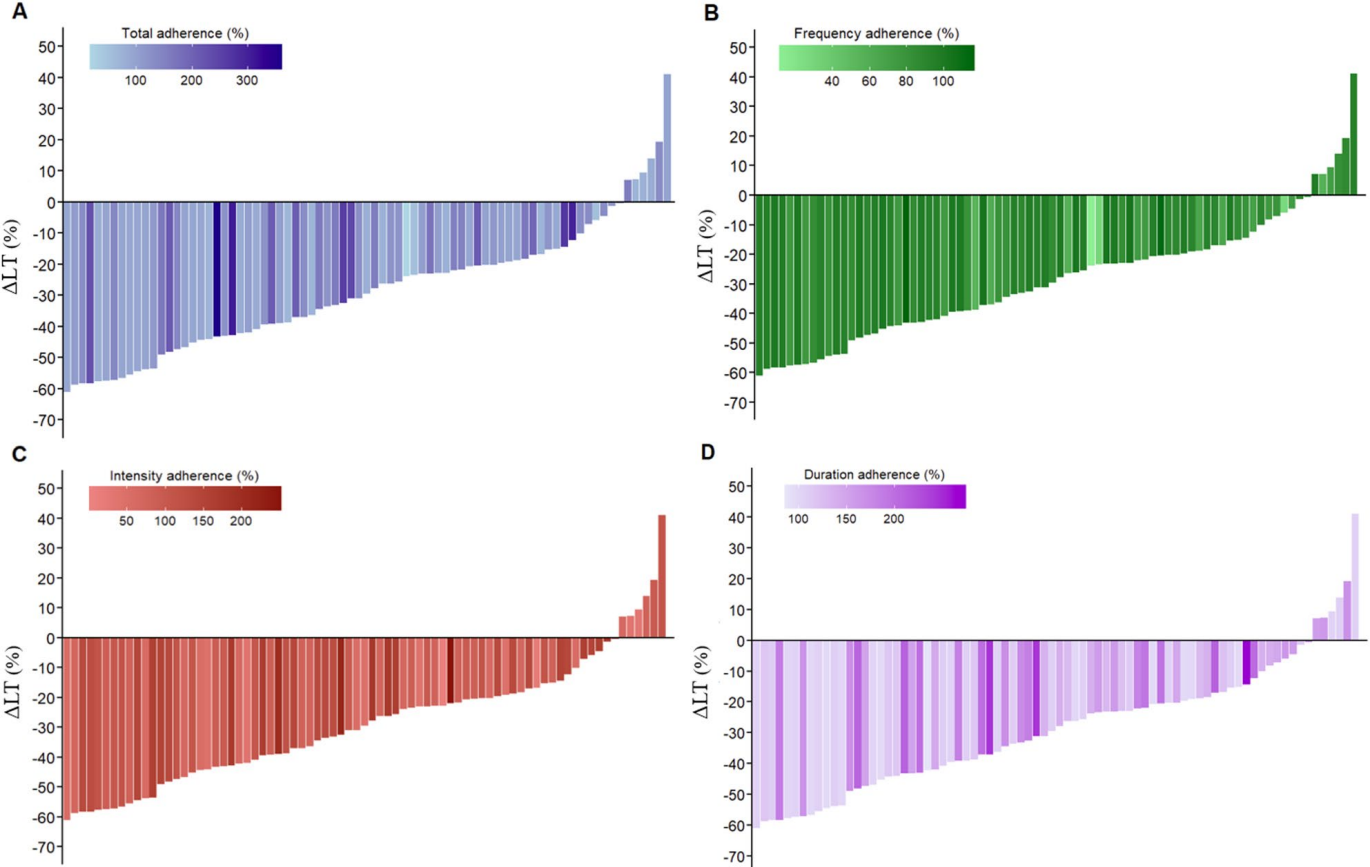
Individual participant percentage changes in LT in response to the six-month exercise intervention, with colors representing each individual’s (**A**) total adherence, (**B**) frequency adherence, (**C**) intensity adherence, and (**D**) duration adherence. Color-bar legends in the top-left of each panel map the gradient to numerical adherence values (%); darker colors indicate higher adherence levels

From pre- to post-intervention, [La−]_b_ at the pre-intervention LT stage decreased from 4.4 mmol/L (95% CI 4.1–4.6) to 3.1 mmol/L (95% CI 2.9–3.3), a mean change of − 1.3 mmol/L (95% CI − 1.5 to − 1.0) or − 27.6% (95% CI − 32.9 to − 22.2%), consistent with a rightward shift of the lactate curve. PLSR analysis found that the adherence metrics did not significantly predict post-test LT. To assess whether a specific adherence metric was associated with post-test LT, multiple linear regression analyses were performed, accounting for baseline LT, age, sex, and country of residence. Similarly to PLSR analyses, these showed no associations between post-test LT and total adherence (β = −0.00, 95% CI: −0.01; 0.00), frequency adherence (β = −0.01, 95% CI: −0.02; 0.00), intensity adherence (β = 0.00, 95% CI: −0.00; 0.01), or duration adherence (β = −0.00, 95% CI: −0.01; 0.01). See Fig. 5 for individual participant changes in %LT in response to the six-month exercise intervention.

### Adverse events

Safety and adverse events were monitored through regular participant–coach communication, which also captured reasons for withdrawal. Twenty-four participants withdrew; none reported exercise-related injury as the primary reason. Five withdrew because they perceived the program as too demanding, 11 due to health issues unrelated to the intervention, six for personal reasons, and two did not report a reason.

Minor musculoskeletal complaints were reported by 18 participants (e.g., transient muscle tightness/strain or exacerbation of pre-existing knee/hip pain). Three participants reported difficulty achieving the prescribed intensity after recovering from COVID-19. Three falls occurred during outdoor interval sessions; one resulted in superficial cuts/scrapes, and two resulted in hairline rib fractures. The rib-fracture incidents occurred on uneven terrain, underscoring the importance of route selection and environmental risk assessment when prescribing outdoor interval training for older adults.

## Discussion

The primary aim of this study was to determine the associations between objectively measured adherence to a home-based exercise intervention and CRF responses, specifically V̇O_2PEAK_ and LT, in older adults, to inform practical exercise prescription and monitoring in real-world settings. The results showed that larger intervention-induced changes in V̇O_2PEAK_ were associated with higher total adherence (cumulative MET-mins), frequency adherence (sessions per week), and intensity adherence (minutes > 80%HR_PEAK_) but not with duration adherence. In contrast, no associations were found between changes in LT and any adherence metrics.

### Associations between adherence and V̇O_2PEAK_

It is reasonable to hypothesize that individuals with superior adherence to exercise training would achieve greater improvements in CRF. Indeed, findings from the present study support this hypothesis, indicating that exercise frequency, intensity, and total volume contribute to V̇O_2PEAK_ gains. Notably, adherence was generally high (overall adherence = 122 ± 59%), with many participants completing more exercise than prescribed, suggesting that it may not be adherence to a specific program structure alone but rather the underlying principles of exercise volume and intensity that are most relevant to V̇O_2PEAK_ improvements. Importantly, this study shows that home-based exercise interventions can be successfully adhered to and are a viable option to increase V̇O_2PEAK_ in older adults. However, the literature remains inconclusive as to whether exercise volume or intensity holds greater importance for CRF adaptations.

Regarding exercise intensity, a meta-analysis by Bouaziz et al. [1] reported superior effects of high-intensity exercise training, as prescribed in the present study, on CRF gains compared to MICT training in older adults. Furthermore, Ross et al. [14] demonstrated that, compared with lower-intensity, six months of higher-intensity exercise, matched for total energy expenditure, resulted in superior V̇O_2PEAK_ gains and a lower rate of non-responders (0% versus 18%) in middle-aged adults. In contrast, our previous meta-analysis indicates that the effect of exercise intensity on CRF gains in older adults is not as strong as commonly assumed [38]. Specifically, there was only a small-to-moderate, non-significant effect of high-intensity training on V̇O_2PEAK_ gains relative to moderate-intensity training after adjusting for total exercise volume. Similarly, Mølmen et al. [39] found that V̇O_2MAX_ improved comparably across endurance training, HIIT, and sprint interval training, with HIIT showing a non-significant tendency for greater improvement. The present findings, however, indicate that older adults who completed more high-intensity exercise (i.e., > 80%HR_PEAK_) experienced superior V̇O_2PEAK_ gains, but adherence to total exercise volume had a greater effect. Taken together, these results highlight the need to disentangle the relative and joint contributions of intensity and volume, particularly in home-based interventions among older adults, and, accordingly, future research should determine the optimal exercise volume and develop approaches to identify individuals who may require higher intensity and/or volume to achieve meaningful adaptations.

Exercise volume was also hypothesized to promote greater CRF gains, which our data support. Our primary adherence metric was the cumulative MET-mins, representing total exercise volume, derived from exercise intensity, duration, and frequency data. Similarly to exercise intensity, cumulative MET-mins and exercise frequency (sessions per week) were positively associated with changes in V̇O_2PEAK_, indicating that participants who completed a greater volume of exercise and exercised more regularly experienced larger V̇O_2PEAK_ gains. Previous research has indicated that adherence to the number of sessions completed has a limited impact on fitness outcomes; however, completing a higher per-session exercise volume (i.e., greater energy expenditure) at a given intensity leads to significantly greater CRF gains and reduces non-responder rates from 38% to 18% [14]. Similarly, Sisson et al. [40] observed a positive effect of increased exercise volume on V̇O_2MAX_ gains in older female adults, though benefits diminished when exercise volume exceeded recommended guidelines, suggesting a possible threshold effect. Furthermore, Montero & Lundby [15] reported a progressive reduction in non-responder rates with higher weekly training volumes (81%, 50%, and 0% for 60, 180, and 300 min per week, respectively), with all non-responders achieving response status by adding two additional weekly sessions. In line with these findings, Mølmen et al. [39] found that higher training frequencies (6 > 4 > 2 sessions per week) were associated with larger increases in V̇O_2MAX_. Collectively, these previous findings highlight the importance of exercise volume for CRF gains and align with the results of the present study.

Although exercise duration varied widely among participants (Table 2), adherence was > 80% for all, with 29 participants completing long sessions (i.e., > 150% duration adherence). These high adherence rates may explain the lack of association with CRF responses, indicating that exercising for longer than the prescribed duration (mean planned duration = 29.5 min) was not beneficial for V̇O_2PEAK_ improvements in untrained individuals.

Taken together, our findings highlight the importance of exercise frequency, intensity, and total volume in achieving V̇O_2PEAK_ gains in untrained older adults. Notably, exercise volume appears to have the greatest effect on V̇O_2PEAK_ gains within this population.

### Associations between adherence and LT

Although adherence metrics appear to be important for V̇O_2PEAK_ gains, the present data indicate that these metrics did not influence changes in LT, and thus other factors beyond adherence may play a larger role in influencing LT adaptations. In contrast, previous research by Dalleck et al. [41] investigated the dose-response relationship between interval training frequency and improvements in LT over six weeks, reporting that physically active participants who completed two additional HIIT sessions per week experienced greater improvements in LT in comparison to one additional session per week. However, these participants were young, physically active adults, which may explain discrepancies with the present findings.

In our previous publication [27], we demonstrated that the magnitude of change in LT was greater than that observed in V̇O_2PEAK_, with all but ten participants showing improvements of > 5% in LT, while 41 participants showed improvements of < 5% in V̇O_2PEAK_. This suggests that even participants with lower-than-prescribed adherence levels experienced gains in LT, which could explain the lack of association observed between adherence metrics and changes in LT. These findings imply that LT may respond more readily to the overall stimulus provided by the exercise intervention, regardless of specific adherence to frequency, intensity, or duration.

Additionally, findings from Bonafiglia et al. [17] support the idea that inter-individual variability plays a significant role in LT adaptations. Their study observed substantial individual differences in responses to endurance and sprint interval training, including V̇O_2PEAK_, LT, and submaximal heart rate, where improvements in V̇O_2PEAK_ were not necessarily associated with improvements in LT. In their study, high non-response rates were observed in V̇O_2PEAK_ (48% in endurance training and 57% in sprint interval training), whereas LT showed a lower non-response rate in endurance training (27%) but a higher non-response rate in sprint interval training (63%).

In contrast, the present study, which utilized a HIIT protocol, demonstrated that LT improvements were more consistent than those in V̇O_2PEAK_. This suggests that LT may be a more trainable metric in older adults, even with high-intensity protocols, and that participants responded to HIIT with more reliable adaptations in LT compared to V̇O_2PEAK_. One potential explanation for this may be the variability in adherence to intensity targets among participants. Notably, 39 participants completed < 80% of the planned high-intensity exercise time, which could mean that for these participants, the actual training stimulus was closer to moderate intensity levels, potentially favoring LT adaptations over V̇O_2PEAK_. Beyond this intensity-stimulus interpretation, a further non-mutually-exclusive explanation warrants consideration. The dose-response curve for LT may show diminishing returns, with much of the adaptive gain realized at relatively low doses of training volume or intensity. If so, even sub-threshold adherers in our cohort may have received sufficient stimulus to drive much of the available LT improvement, attenuating the variance in ΔLT available for adherence metrics to explain. This interpretation is consistent with the near-universal LT improvement previously observed in this cohort [27].

Collectively, our findings indicate that LT improvements may be less dependent on specific adherence metrics and more responsive to the overall stimulus provided by exercise training, making it a valuable outcome measure in exercise interventions for older adults.

### Strengths and limitations

A key strength of this study is the use of objective, session-level adherence data collected throughout a 26-week, home-based intervention, with heart-rate traces quality-checked and adherence quantified using multiple FITT-derived metrics, including a MET-based estimate of total exercise volume (cumulative MET-mins) derived from each session’s duration and mean %HR_PEAK_. In addition, V̇O_2PEAK_ and LT were assessed using a standardized graded treadmill protocol with continuous respiratory gas measurements and blood lactate sampling, and multivariable associations were examined using cross-validated PLSR to address multicollinearity among adherence metrics.

However, the participants in the present study were older adults aged 60–84 years who did not regularly exercise before entering the study, yet were relatively healthy and physically active [27]. This specificity may limit the generalizability of the findings to broader populations, particularly those with greater functional impairments, lower physical activity levels, or those in the ‘oldest-old’ category. Furthermore, it should be considered that associations may not be strictly linear, with a potential upper limit to the beneficial effects, or even detrimental effects, at excessive exercise. In addition, the adherence metrics in the present study were tailored to quantifying compliance with the specific home-based HIIT prescription used (i.e., adherence “within” a single training model). Because the intervention did not include a moderate- or low-intensity comparator (e.g., MICT), we cannot determine whether the observed adherence–response relationships are specific to HIIT or reflect more general dose–response effects across a broader range of training intensities. Cumulative MET-min should not be interpreted as a fully intensity-independent measure of exercise dose, since its calculation incorporates session %HR_PEAK_ as the intensity weighting. Relatedly, although some participants trained at lower effective intensities due to difficulty meeting the > 80% HR_PEAK_ target, this did not provide a controlled intensity contrast and may have limited our ability to isolate the relative contribution of prescribed intensity versus overall volume. Variation in the achieved exercise intensity, and the fact that some participants exercised longer than the prescribed minimum duration, are inherent features of an unsupervised home-based intervention and should be considered when interpreting the adherence-response associations reported here. Two further limitations should be acknowledged. The intervention overlapped with the tail-end of the COVID-19 pandemic, and we cannot exclude broader residual influences on adherence patterns. Additionally, because V̇O_2PEAK_ and LT were assessed by open-laboratory maximal exercise testing, outcome assessors could not be blinded to group assignment, which we acknowledge as a potential source of assessor bias.

## Conclusions

This study aimed to investigate the associations between objectively measured adherence to a six-month home-based exercise intervention and the responses of V̇O_2PEAK_ and LT in older adults. Results showed that changes in V̇O_2PEAK_ were positively associated with total, frequency, and intensity adherence, with the strongest predictive contribution observed for total adherence. In contrast, adherence metrics were not associated with changes in LT. Taken together, the results indicate that overall exercise volume (total adherence; cumulative MET-min) is the adherence component most closely linked to V̇O₂_PEAK_ improvements in this home-based HIIT program, with frequency and intensity also contributing positively.

### Practical implications

The findings of this study provide insights into designing effective exercise interventions for older adults. Programs aiming to improve CRF, particularly V̇O_2PEAK_, may benefit from emphasizing strategies that support achieving a high overall exercise volume, as total adherence (cumulative MET-mins) showed the strongest predictive contribution to V̇O_2PEAK_ improvements in this cohort. While exercise frequency and intensity also showed positive associations, their influence was weaker, suggesting that maintaining overall exercise volume may be particularly important for improving CRF. This emphasis on volume may be especially relevant for individuals who find high-intensity exercise (> 80% HR_PEAK_) challenging, offering a viable alternative pathway to improvement. However, as LT improvements were found to occur across varying adherence levels, interventions may consider a more versatile approach for LT-focused training, allowing participants to achieve gains with different prescriptions of the FITT principle. These insights suggest that individualized training programs that account for both adherence and individual variability could enhance CRF and ensure more accessible, adaptable programs for older adults engaging in home-based exercise. Importantly, future research should aim to objectively assess adherence, as variations in adherence are likely to influence responses to outcome measures, such as CRF. Without accurate adherence data, it becomes difficult to draw strong conclusions about the observed effects. Lastly, future work with larger samples should examine whether adherence–response relationships differ by sex, with particular attention to postmenopausal females, in whom sex-specific responses to home-based HIIT remain incompletely characterized.

## Data Availability

All relevant data have been uploaded and are accessible via OSF Storage (https:/osf.io/d7aw2).

## Abbreviations

ACSM: American College of Sports Medicine
BMI: Body mass index
CI: Confidence interval
CRF: Cardiorespiratory fitness
ECG: Electrocardiogram / electrocardiography
FAB: Fitness, Ageing, and Bilingualism (project)
FITT: Frequency, intensity, time, and type
HIIT: High-intensity interval training
HR_PEAK_: Peak heart rate
LT: Lactate threshold
MET: Metabolic equivalent of task
MICT: Moderate-intensity continuous training
OSF: Open Science Framework
PLSR: Partial least squares regression
SPSS: Statistical Package for the Social Sciences (IBM SPSS Statistics)
V̇CO_2_: Carbon dioxide output
V̇O_2_: Oxygen uptake
V̇O_2PEAK_: Peak oxygen uptake
